# A study of the association between the early pregnancy thrombosis–thyroid axis and pregnancy outcomes in patients with recurrent spontaneous abortion

**DOI:** 10.3389/fendo.2026.1890736

**Published:** 2026-07-29

**Authors:** Yidi Wang, Jiarun Zhao, Guanshan Li, Shuchang Zheng, Chengxia Wang, Chanjuan Sang, Qian Han, Mengyuan Li, Junqin He

**Affiliations:** Department of Traditional Chinese Medicine, Beijing Obstetrics and Gynecology Hospital, Capital Medical University. Beijing Maternal and Child Health Care Hospital, Beijing, China

**Keywords:** coagulation-fibrinolysis system, platelet aggregation, pregnancy outcome, recurrent spontaneous abortion, thrombophilia, thyroid function

## Abstract

**Background:**

Recurrent spontaneous abortion (RSA) is a severe, yet common, pregnancy complication. Thrombophilia and thyroid dysfunction are critical factors altering placental microcirculation and embryonic development, although the synergistic predictive value of these two conditions for RSA pregnancy outcomes remains undetermined.

**Objective:**

To investigate the factors influencing pregnancy outcomes in patients with RSA; analyze the association between early pregnancy markers (including coagulation and fibrinolysis, platelet function, and thyroid function) and pregnancy outcomes in patients with RSA; identify independent risk factors and evaluate their predictive performance; explore the association between thrombotic markers and thyroid function parameters in the RSA population; elucidate the regulatory mechanisms of the thrombosis–thyroid function axis; and provide evidence-based support for early clinical risk prediction.

**Methods:**

We included 400 patients with RSA who were enrolled between January 2022 and December 2024 after screening and categorized them into successful pregnancy and pregnancy loss groups. Baseline clinical data and 20 early pregnancy laboratory parameters were collected. Univariate and multivariate logistic regression analyses were performed to screen risk factors. Spearman correlation analysis was used to assess thrombotic–thyroid axis correlations, and receiver operating characteristic (ROC) curves were adopted to evaluate the predictive value of relevant indicators.

**Results:**

In intergroup comparison, the pregnancy loss group exhibited significantly higher antithrombin III (AT), protein C (PC), protein S (PS), thrombin time (TT), adenosine diphosphate (ADP), arachidonic acid (AA), free thyroxine (FT4), and thyroid-stimulating hormone (TSH) levels (P < 0.05), whereas the successful pregnancy group had higher D-dimer (DD), fibrinogen (FIB), and prothrombin activity (PA) levels (P < 0.05). Significant intergroup differences were not observed in activated partial thromboplastin time (APTT), prothrombin time (PT), free triiodothyronine (FT3), homocysteine (HCY), and antinuclear antibodies (ANA). Multivariate logistic regression demonstrated that elevated PS, increased ADP/AA-induced platelet aggregation rate, elevated FT4 and TSH, and reduced DD were independent risk factors for recurrent pregnancy loss (P < 0.05). Thrombotic markers and thyroid function markers showed widespread moderate-to-strong positive correlations in the successful pregnancy and pregnancy failure groups; however, cross-system synergistic correlations were significantly weaker in the pregnancy failure group, with correlation coefficients between AT and TSH of 0.19 and −0.0573, respectively, and a P-value of 0.013 for the between-group comparison (P < 0.05). The ROC analysis exhibited that the area under the curve of the single DD indicator for predicting pregnancy loss was 0.591 (95% confidence interval: 0.536–0.647), showing low-to-moderate predictive efficacy.

**Conclusion:**

There is a certain correlation between coagulation and thyroid function, with differences existing among patients with recurrent miscarriage but different pregnancy outcomes. Combined testing of PS, DD, ADP, AA, FT4, and TSH during early pregnancy may provide supplementary information for risk assessment in individuals with RSA.

## 1 Introduction

Recurrent spontaneous abortion (RSA) refers to ≥2 consecutive pregnancy losses with the same sexual partner before 28 weeks of gestation, including adverse outcomes such as spontaneous abortion, embryonic arrest, and biochemical pregnancy. Its incidence rate is approximately 1%–5% (1, 2), showing a substantial upward trend with advancing maternal age and an increasing number of previous abortions (3). RSA can cause long-term complications such as endometrial damage, endocrine disorders, and pelvic infections, as well as psychological disorders, including anxiety and depression, placing a heavy burden on families and society. Thus, it has become a major public health issue in reproductive health.

The etiology of RSA involves the interaction of multiple factors, including genetic, anatomical, endocrine, autoimmune, infectious, and thrombophilia factors. Among these, embryonic chromosomal abnormalities account for > 50% of early miscarriages, whereas uterine anatomical abnormalities increase the risk of miscarriage by interfering with embryonic implantation and development (4). Despite the ongoing in-depth etiological research, the underlying cause remains undetermined in 40%–50% of RSA cases, complicating clinical management and considerably increasing the risk of recurrent pregnancy loss (5). Unlike in infertility, the management of which focuses on achieving pregnancy, the core clinical objectives of RSA treatment are to maintain pregnancy to term, reduce the miscarriage rate, and increase the live birth rate. Therefore, early identification of risk factors and the implementation of stratified interventions are crucial.

A prothrombotic state, characterized by an imbalance in the anticoagulant–fibrinolytic system and hypercoagulability, can trigger microthrombosis in the placental microcirculation, leading to inadequate placental perfusion, embryonic ischemia and hypoxia, and thereby subsequent pregnancy loss (6). Moreover, thyroid dysfunction considerably increases the risk of adverse pregnancy outcomes by disrupting endocrine homeostasis, affecting luteal function and placental hormone synthesis, and compromising maternal–fetal immune tolerance (7). Early pregnancy markers, such as coagulation function, platelet aggregation, thyroid function, homocysteine, and antinuclear antibodies, represent routine non-invasive obstetric tests that can comprehensively assess the mother’s pathophysiological status (8).

Currently, clinical practice often relies on single markers such as progesterone and human chorionic gonadotropin (hCG) to assess the risk of pregnancy failure, which provide limited predictive efficacy and lack a multidimensional, multisystem integrated assessment system (9). Based on a single-center retrospective cohort, in this study, we systematically analyze the association between early pregnancy indicators—including coagulation–fibrinolysis, platelet function, and thyroid function—and subsequent pregnancy outcomes. Additionally, we aimed to identify independent risk factors and investigate the association between thrombosis-related markers and thyroid function parameters in patients with RSA, thereby providing high-quality, evidence-based data to support early risk assessment and precision diagnosis and treatment for patients with RSA.

## 2 Materials and methods

### 2.1 Study design

This single-center retrospective cohort study, conducted at the Beijing Obstetrics and Gynecology Hospital, Capital Medical University, was approved by the Ethics Committee of the Beijing Obstetrics and Gynecology Hospital, Capital Medical University (approval No.: 2025-KY-080-01), with an exemption from obtaining patients’ informed consent. All procedures followed medical ethical standards.

### 2.2 Study population

Patients with RSA who visited the Department of Traditional Chinese Medicine at Beijing Obstetrics and Gynecology Hospital, Capital Medical University, between January 2022 and December 2024 were included in the study. RSA was defined as ≥2 consecutive pregnancy losses (including biochemical pregnancies) before 28 weeks of gestation with the same sexual partner. Based on the outcome of the subsequent pregnancy, patients were divided into two groups: the successful pregnancy group (RSA-L), defined as a current pregnancy reaching ≥28 weeks with a live birth (n = 200); and the pregnancy failure group (RSA-F), defined as a current pregnancy ending in spontaneous abortion, embryonic arrest, or biochemical pregnancy before 28 weeks (n = 200).

Patients who (1) met the diagnostic criteria for recurrent miscarriage, (2) were aged ≥18 years, and (3) had a subsequent pregnancy in the first trimester (≤12 weeks of gestation) were included. Exclusion criteria comprised (1) patients in the acute phase of cardiovascular, cerebrovascular, hepatic, renal, or hematopoietic system diseases; (2) organic or structural abnormalities of the reproductive tract; and (3) recurrent miscarriage caused by chromosomal abnormalities or genetic disorders in either or both partners.

### 2.3 Sample size estimation

Based on previous studies, the effect size d for the key indicators between the RSA failure and success groups was approximately 0.4. With α set at 0.05 and a power of 1−β = 0.80, a two-sample mean comparison required at least 100 cases per group. For the multivariate logistic regression, we planned to include 10–12 independent variables. We determined that 200 cases per group, for a total sample size of 400, would meet the statistical power requirements since the number of events should be 10–15 times the number of independent variables. The study employed a 1:1 balanced grouping (200 cases per group), as this balanced-sample design optimizes the statistical power of the tests and ensures balance in baseline characteristics between the groups. This study is an interim analysis of a large clinical cohort: we statistically analyzed 400 eligible cases while continuing the enrollment of cases into the large cohort.

### 2.4 Data collection

Baseline data collected were age, body mass index (BMI), number of miscarriages, smoking history, alcohol consumption history, menstrual history, and obstetric history. For laboratory parameters, fasting venous blood samples were collected between 10 and 12 weeks of early pregnancy, uniformly tested by the hospital’s laboratory using standardized instruments and reagents, comprising 20 items: (1) coagulation parameters: fibrinogen (FIB), prothrombin time (PT), international normalized ratio (INR), prothrombin activity (PA), thrombin time (TT), activated partial thromboplastin time (APTT), and D-dimer (DD); (2) thrombotic risk markers: antithrombin III (AT), protein C (PC), and protein S (PS); (3) platelet aggregation: adenosine diphosphate (ADP) and arachidonic acid (AA); (4) metabolic markers: homocysteine (HCY), vitamin A (VitA), vitamin E (VitE), and 23-hydroxyvitamin D (23-OH-VD); (5) thyroid function: free triiodothyronine (FT3), free thyroxine (FT4), thyroid-stimulating hormone (TSH); (6) autoimmunity: antinuclear antibodies (ANA).

### 2.5 Statistical analysis

Data analysis was performed using SPSS 26.0 software. Continuous variables are expressed as mean ± standard deviation or median (25th percentile, 75th percentile). The groups were compared using the independent samples t–test or the Mann–Whitney U test. Categorical variables are expressed as percentages (%), using the chi-square (χ²) test. Variables with P-value < 0.05 in univariate analysis were included in a multivariate logistic regression model to identify independent risk factors. Spearman’s correlation analysis was used to assess the association between thrombotic markers and thyroid function indicators. ROC curves were plotted to evaluate the predictive value of the indicators, and the area under the curve (AUC) and 95% confidence interval (CI) were calculated. A P-value < 0.05 was considered statistically significant.

## 3 Results

### 3.1 Subject selection process

**Figure 1:**
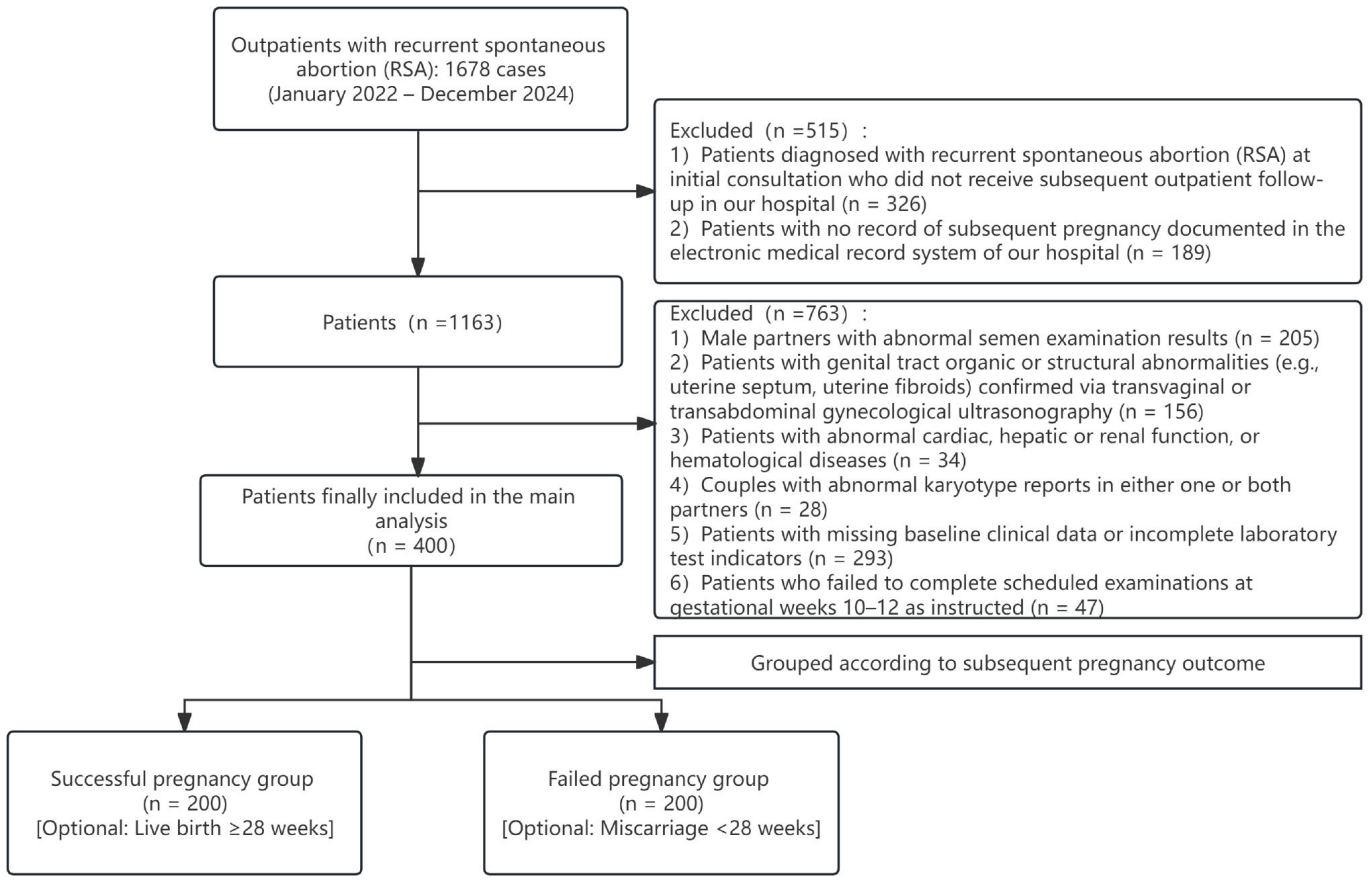
Flowchart of patient enrolment and grouping.

A total of 1,678 patients with RSA were initially screened. After stratified screening using inclusion and exclusion criteria, 400 patients were included in the statistical analysis, with 200 patients in each group. **Figure 1** demonstrates the screening process.

### 3.2 Comparison of baseline and laboratory parameters between the two groups

There were no statistically significant differences in general characteristics such as age and BMI between the two groups (P > 0.05); hence, the groups were well-balanced and comparable at baseline.

Among the 200 pregnancies in the successful pregnancy group, 4.60% resulted from assisted reproductive technology, while 95.40% resulted from natural conception. The neonatal survival rate was 100%. Furthermore, 19.54%, 80.46%, and 0% of deliveries occurred between 28 and 37 weeks, between 37 and 42 weeks, and at 42 weeks of gestation or later. Additionally, 38.51% of patients had vaginal deliveries, and 61.49% of patients had cesarean sections, with no major perinatal complications observed. In the pregnancy failure group (200 cases), biochemical pregnancy accounted for 4.22%, early miscarriage accounted for 92.97%, mid-term miscarriage accounted for 2.81%, and late miscarriage accounted for 0%. Due to the limitations of this retrospective study, follow-up and treatment outcomes were not included in the statistical analysis.

The comparison of coagulation and thrombophilia markers showed that AT, PC, PS, and TT levels were significantly higher in the pregnancy failure group than in the successful group (P < 0.05). Conversely, FIB, PA, and DD levels were significantly higher in the successful group (P < 0.05). APTT, PT, or INR demonstrated no differences between the groups (P > 0.05). Regarding platelet function, the pregnancy failure group showed significantly higher ADP- and AA-induced platelet aggregation rates (P < 0.001). The comparison of thyroid function revealed that FT4 and TSH levels were significantly higher in the pregnancy failure group than in the successful group (P < 0.05), and FT3 showed no differences between the groups (P > 0.05). In the successful pregnancy group, 6 patients had TSH levels > 4 mIU/L, 162 patients had TSH levels < 2.5 mIU/L, 1 patient had subclinical hyperthyroidism, 1 patient had clinical hypothyroidism, and 5 patients had subclinical hypothyroidism. In the pregnancy failure group, 22 patients had TSH levels > 4 mIU/L, 139 had TSH levels < 2.5 mIU/L, 10 had clinical hypothyroidism, and 12 had subclinical hypothyroidism. All of the above diagnoses were made according to the criteria for early pregnancy outlined in the “Guidelines for the Prevention and Management of Thyroid Diseases During Pregnancy and Childbirth” (10). However, there were no statistically significant differences between groups for HCY, VitA, VitE, 23-OH-VD, or ANA (P > 0.05; **Table 1**).

**Table 1:** Comparison of general information characteristics and laboratory indicators of patients with RSA-L and RSA-F.

Characteristic	RSA-L (n=200)	RSA-F (n=200)	t/Z	P value
Age (years)	33.96±3.794	33.70±3.698	0.703	0.483
BMI (kg/m2)	22.97±3.629	22.30±3.786	1.810	0.071
AT (%)	97.96±13.06	102.02±10.74	-3.408	0.001
PC (%)	108.00 (98.00,123.00)	113 (103.00,128.75)	-2.594	0.009
PS (%)	82.20±27.44	95.53±17.44	-5.794	<0.001
FIB (g/L)	3.42 (2.91,3.98)	3.02 (2.61,3.64)	-4.419	<0.001
PT (s)	11.40 (11.00,11.80)	11.52 (11.09,12.10)	-2.034	0.042
INR (-)	0.96 (0.91,1.01)	0.97 (0.91,1.05)	-1.526	0.127
PA (%)	106.10 (100.00,112.40)	102.15 (95.80,110.85)	-2.621	0.009
TT (s)	16.30 (15.80,16.90)	16.60 (15.73,17.40)	-1.989	0.047
APTT (s)	26.55 (25.40,28.00)	26.70 (25.60,28.50)	-1.501	0.133
DD (μg/mL)	0.31 (0.21,0.70)	0.26 (0.19,0.45)	-3.175	0.002
VitA (mg/L)	0.44 (0.36,0.57)	0.43 (0.36,0.56)	-0.170	0.865
VitE (mg/L)	11.99 (9.65,14.63)	12.29 (10.02,15.01)	-1.100	0.271
23-OH-VD (ng/mL)	21.22 (14.96,28.76)	21.82 (17.36,28.06)	-1.396	0.162
ADP (%)	78.75 (64.58,89.30)	85.25 (76.08,91.90)	-3.933	<0.001
AA (%)	91.10 (85.83,93.78)	93.20 (89.60,95.98)	-4.544	<0.001
HCY (μmol/L)	7.50 (6.46,9.00)	7.70 (6.70,9.01)	-1.365	0.172
FT3 (pmol/L)	4.73 (4.50,5.09)	4.83 (4.53,5.16)	-1.767	0.077
FT4 (pmol/L)	15.54 (13.88,16.70)	15.95 (14.26,18.97)	-2.713	0.007
TSH (mIU/L)	1.52 (0.89,2.20)	1.95 (1.39,2.62)	-4.757	<0.001
ANA (-)	2.35 (1.32,4.68)	2.37 (1.21,5.52)	-0.518	0.605

RSA-L, RSA patients with live birth (successful subsequent pregnancy); RSA-F, RSA patients with failed subsequent pregnancy (miscarriage).

### 3.3 Univariate and multivariate logistic regression analysis

Using pregnancy success in patients with RSA as the dependent variable (pregnancy = 1, no pregnancy = 0), univariate logistic regression analyses were performed on each potential influencing factor. The results showed that elevated AT, PC, PS, APTT, ADP, AA, FT4, TSH, and HCY levels were risk factors for pregnancy failure, while elevated DD levels were a protective factor (all P < 0.05; **Table 2**).

**Table 2:** Univariate logistic regression analysis of factors associated with pregnancy outcome in patients with RSA.

Characteristic	B	SE	Wald	P	Exp (B)	95%CI
ANA	0.008	0.006	1.701	0.192	1.008	0.996,1.019
AT	0.030	0.009	10.942	0.001	1.030	1.012,1.049
PC	0.011	0.005	4.830	0.028	1.011	1.001,1.020
PS	0.026	0.005	28.532	<0.001	1.027	1.017,1.037
FIB	-0.099	0.065	2.309	0.129	0.906	0.798,1.029
PT	0.033	0.039	0.701	0.402	1.033	0.957,1.116
INR	1.254	0.854	2.157	0.142	3.503	0.657,18.665
PA	-0.014	0.009	2.351	0.125	0.986	0.968,1.004
TT	0.103	0.081	1.629	0.202	1.109	0.946,1.300
APTT	0.108	0.046	5.548	0.019	1.114	1.018,1.219
DD	-0.527	0.163	10.450	0.001	0.590	0.429,0.813
HCY	0.117	0.043	7.430	0.006	1.124	1.033,1.223
ADP	0.036	0.008	17.828	<0.001	1.036	1.019,1.054
AA	0.018	0.005	15.121	<0.001	1.018	1.009,1.027
VitA	0.370	0.347	1.133	0.287	1.447	0.733,2.859
VitE	0.034	0.024	2.006	0.157	1.034	0.987,1.084
23-OH-VD	0.011	0.009	1.380	0.240	1.011	0.993,1.030
FT3	0.364	0.158	5.280	0.022	1.439	1.055,1.964
FT4	0.043	0.016	7.684	0.006	1.044	1.013,1.076
TSH	0.391	0.090	18.651	<0.001	1.478	1.238,1.764

Univariate logistic regression analysis was performed to screen the potential risk factors for adverse pregnancy outcome in RSA patients. SE, standard error; Wald, Wald statistic; OR, odds ratio; CI, confidence interval.

Using pregnancy outcome in patients with RSA as the dependent variable (pregnancy = 1, no pregnancy = 0), we analyzed variables AT, PC, PS, APTT, ADP, AA, FT3, FT4, TSH, HCY, and DD—which were statistically significant in univariate analysis—as independent variables and included them in a multivariate logistic regression model. After diagnosing multicollinearity, the variance inflation factors for all independent variables were <5, indicating no serious multicollinearity issues. The model’s goodness of fit was good according to the Hosmer–Lemeshow test (χ² = 8.219, P = 0.412). After the adjustment for confounding factors, elevated PS, ADP and AA, and FT4 and TSH were independent risk factors for recurrent pregnancy loss in patients with RSA (all P < 0.05), while increased DD was an independent protective factor (**Table 3**). This analysis suggests that an imbalance in the coagulation–fibrinolysis system, excessive platelet activation, and thyroid dysfunction may be independent pathological risk factors for subsequent pregnancy failure in patients with RSA.

**Table 3:** Multivariable logistic regression analysis to identify independent predictors of pregnancy outcome in women with RSA.

Characteristic	B	SE	Wald	Degrees of freedom	P	Exp(B)	95%CI
AT	0.015	0.011	1.882	1	0.170	1.015	0.994,1.037
PC	0.006	0.006	1.232	1	0.267	1.006	0.995,1.018
PS	0.019	0.006	12.357	1	<0.001	1.020	1.009,1.031
APTT	0.067	0.053	1.606	1	0.205	1.070	0.964,1.187
DD	-0.475	0.170	7.849	1	0.005	0.622	0.446,0.867
ADP	0.033	0.010	11.082	1	0.001	1.033	1.013,1.053
AA	0.012	0.005	5.597	1	0.018	1.012	1.002,1.022
HCY	0.099	0.052	3.586	1	0.058	1.104	0.997,1.224
FT3	0.248	0.178	1.946	1	0.163	1.282	0.904,1.818
FT4	0.049	0.017	8.265	1	0.004	1.050	1.016,1.085
TSH	0.336	0.099	11.543	1	0.001	1.399	1.153,1.699
Constant	-12.556	2.238	31.488	1	<0.001	<0.001	

Multivariate logistic regression analysis was performed to identify independent risk factors for adverse pregnancy outcome in RSA patients, with variables showing P<0.05 in univariate analysis included in the model. SE, standard error; Wald, Wald statistic; OR, odds ratio; CI, confidence interval.

### 3.4 Analysis of the relationship between thrombosis and the thyroid axis

**Figure 2:**
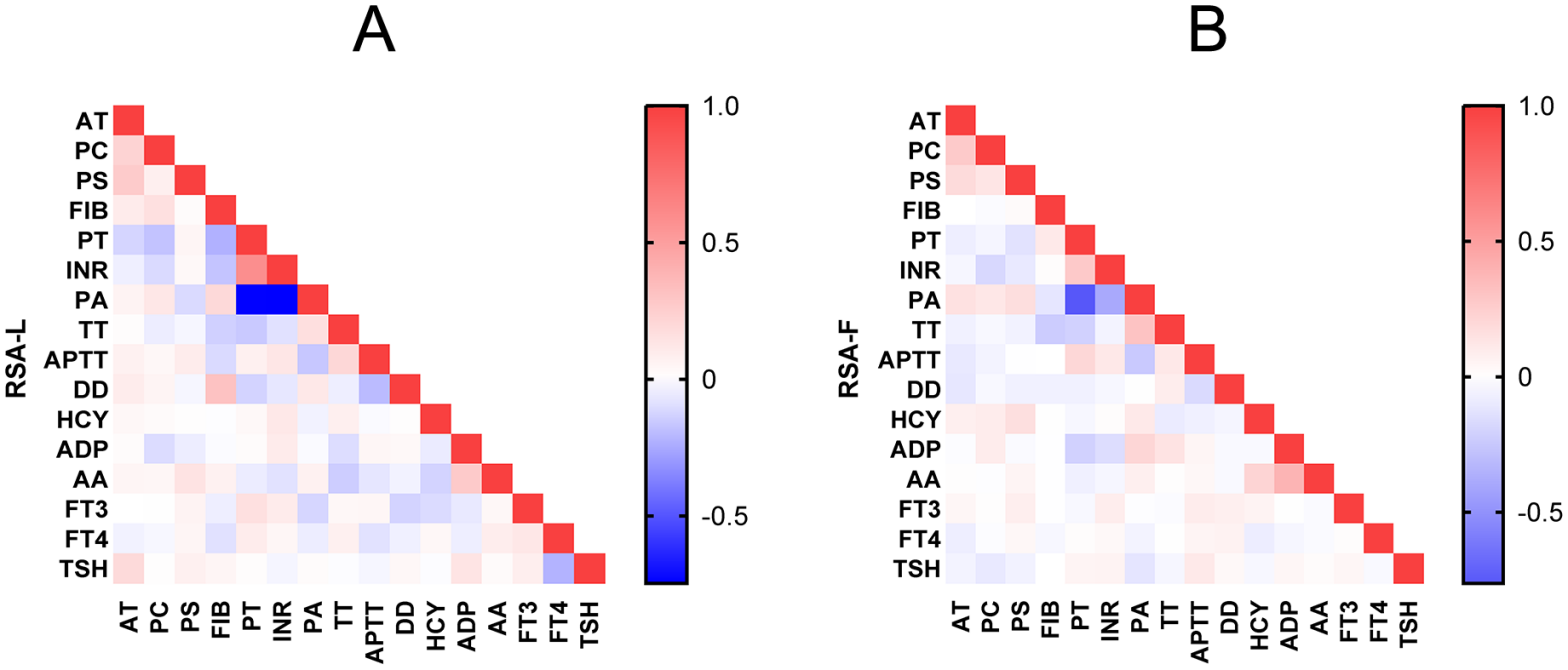
Spearman correlation heatmap of thrombotic markers and thyroid function indicators between the two groups. The color gradient represents the magnitude of the Spearman correlation coefficient (r), ranging from -1 (strong negative correlation, dark blue) to 1 (strong positive correlation, dark red), with 0 indicating no correlation. **(A)** RSA-L, recurrent spontaneous abortion patients with successful subsequent pregnancy (live birth); **(B)** RSA-F, recurrent spontaneous abortion patients with failed subsequent pregnancy (miscarriage).

This study conducted a Spearman correlation analysis of thrombosis-related markers and thyroid function indicators to further investigate the association between pregnancy outcomes and the thrombosis–thyroid axis in RSA. The analysis showed certain correlations with thrombotic and thyroid function markers in the RSA-L and RSA-F groups. Thrombotic markers showed a positive correlation with thyroid function markers (such as AT, ADP, and TSH) in the RSA-L group. In contrast, most markers showed a negative correlation in the RSA-F group, and the strength of these correlations was weaker, with only PA and TSH exhibiting a weak correlation. Unfortunately, the differences in the correlations between the two groups cannot completely rule out the influence of confounding factors (**Figure 2**, **Table 4**).

**Table 4:** Comparison of Spearman correlations between thrombotic-related indicators and thyroid function indicators in the two groups.

Characteristic	RSA-L TSH		RSA-F TSH	
	r	p	r	p
AT	0.19	0.007	-0.0573	0.42014
PC	0.007	0.922	-0.104	0.14146
PS	0.074	0.297	-0.060	0.395
FIB	0.047	0.504	-0.003	0.971
PT	0.015	0.838	0.053	0.460
INR	-0.030	0.674	0.059	0.404
PA	0.020	0.779	-0.122	0.085
TT	-0.010	0.883	-0.042	0.557
APTT	-0.028	0.697	0.112	0.116
DD	0.037	0.604	0.032	0.655
HCY	-0.010	0.892	-0.038	0.589
ADP	0.138	0.051	0.045	0.528
AA	0.023	0.742	0.017	0.811
FT3	0.088	0.216	0.043	0.542
FT4	-0.224	0.001	-0.030	0.672

For example, r for AT and TSH was 0.19 (P = 0.007) in the successful group and −0.0573 (P = 0.420) in the unsuccessful group. The intergroup comparison yielded Z = 2.478 and P = 0.013, indicating a statistically significant difference. Therefore, a certain correlation between thrombotic markers and thyroid function exists, which may be one of the reasons for recurrent pregnancy loss in patients with RSA (**Table 5**).

**Table 5:** Comparison of the correlation between AT and TSH in the two groups.

Group/r	n	TSH/AT
RSA-L	200	0.190
RSA-F	200	-0.057
Z		2.478
P		0.013

### 3.5 Analysis of the predictive value of risk factors

**Figure 3:**
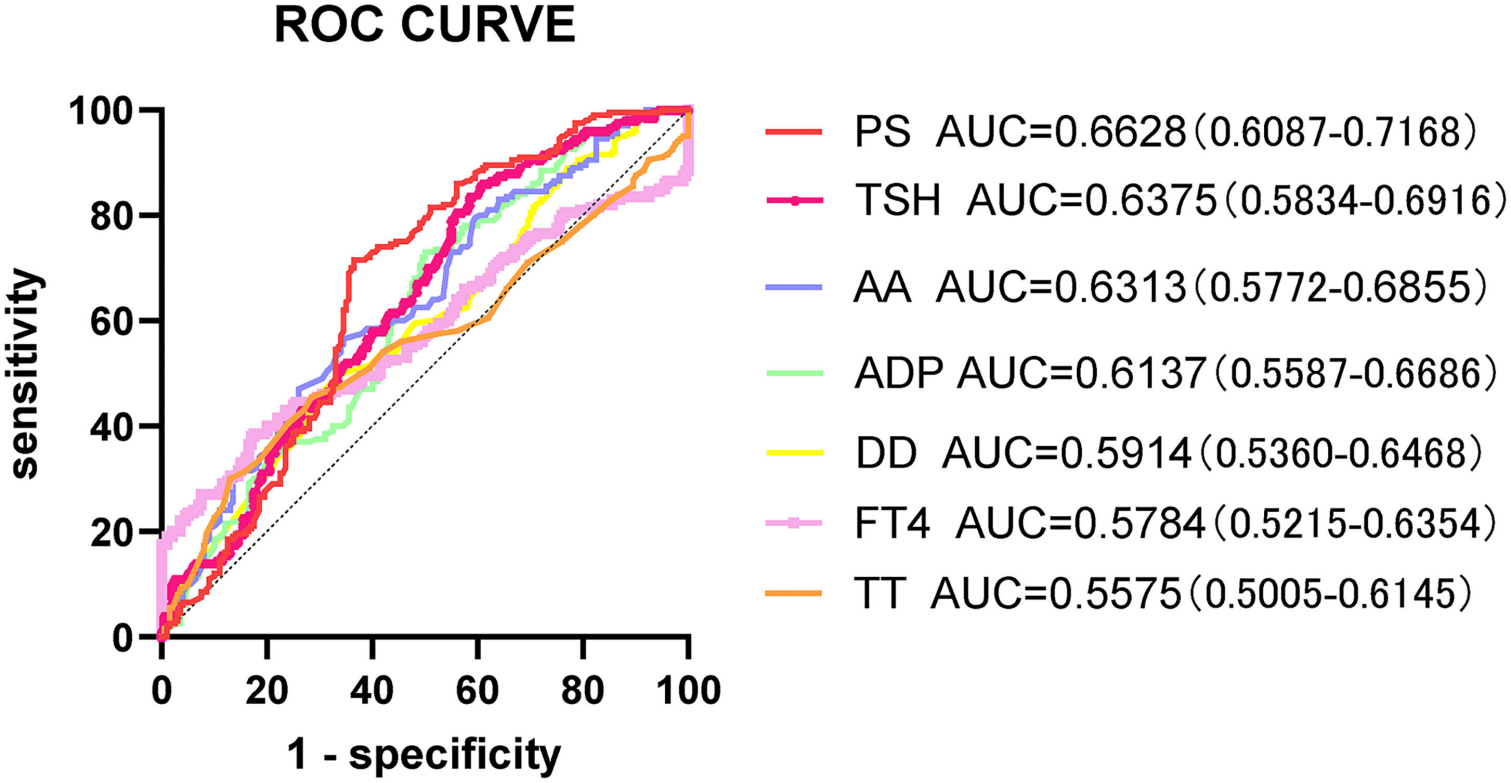
ROC curves of various indicators for predicting pregnancy failure in patients with RSA.

When including PS, TT, DD, ADP, AA, FT4, and TSH in the ROC analysis, the AUC for PS for predicting pregnancy failure was 0.6628 (95% CI: 0.6087–0.7168). As a single marker, it demonstrated low-to-moderate predictive performance, suggesting that combined testing of multiple markers could enhance the predictive value (**Figure 3**).

## 4 Discussion

Patients with RSA often experience physical and psychological stress due to their past miscarriages. With a new pregnancy, waiting until substantial abnormalities in test results become identifiable to intervene often makes it difficult to preserve the pregnancy, leading to the regret of another pregnancy loss. Therefore, the way to identify and intervene in the physical condition of these patients as early as possible—particularly by strengthening the early diagnosis and assessment of abnormalities related to thrombophilia and thyroid dysfunction in early pregnancy—is a critical clinical practice issue that warrants urgent resolution.

In this single-center retrospective study, we included 400 patients with RSA in early pregnancy. We systematically analyzed the association between 20 parameters (including coagulation and fibrinolysis, platelet aggregation, and thyroid function markers) and the outcome of subsequent pregnancies and explored the interrelationship between thrombotic markers and thyroid function. In this study, we aimed to provide evidence-based laboratory criteria for the risk stratification of adverse pregnancy outcomes in early pregnancy among patients with RSA. The results showed substantial differences between the successful and unsuccessful pregnancy groups in some thrombotic and thyroid function markers, which also showed a certain correlation. Multivariate regression identified several independent risk factors. Despite the limited predictive power of individual biomarkers, the combined assessment of multiple markers demonstrated clinical value.

### 4.1 Analysis of the mechanisms linking thrombosis-related markers to pregnancy outcomes in women with RSA

Pregnant women usually gradually shift toward a procoagulant state throughout pregnancy, which is reflected in coagulation and fibrinolysis markers as enhanced coagulation and fibrin turnover (11, 12). We observed that the group with a successful pregnancy exhibited lower AT, PC, and PS levels, shorter TT, and higher FIB, PA, and DD levels compared to the group with pregnancy failure. This differs from the typical pattern observed in infertile populations with traditional thrombophilia, characterized by “decreased anticoagulants and increased procoagulants.” The presumed reason may be that the measurements were obtained during early pregnancy, with all participants in the normal gestational phase. AT, PC, and PS values in both groups essentially met the minimum standards (referring to our hospital’s reference ranges), while FIB, PA, and DD were all within normal limits. In a normal pregnancy, the body develops a moderate hypercoagulable state; thus, anticoagulant protein and antithrombin levels are physiologically reduced, while the coagulation–fibrinolysis system remains in balance. Additionally, fibrinogen and D-dimer become physiologically elevated to ensure the initial establishment of this hypercoagulable state. In contrast, the group with pregnancy failure exhibited higher AT, PC, and PS levels, a longer TT, and reduced FIB, PA, and DD levels, indicating that the physiological hypercoagulable state was not adequately established in this group. This may be due to the depletion of the coagulation–fibrinolysis system resulting from a long-term, chronic hypercoagulable state. Chandra et al. (13, 14) found that PT, TT, and APTT were substantially reduced in pregnant animals and humans compared to the non-pregnant state, while AT activity decreased, and FIB and DD levels increased. This is consistent with the characteristics observed in the successful pregnancy group in this study (reduced AT, shortened TT, elevated FIB, and increased DD), further validating the physiological adaptive changes in the coagulation–fibrinolysis system during normal pregnancy.

Multivariate logistic regression analysis demonstrated that after adjusting for confounding factors, elevated PS levels, increased ADP and AA levels, and decreased DD levels were independent risk factors for pregnancy loss. Therefore, markers of prothrombotic status should be closely monitored while managing patients with RSA to enable early risk identification and timely intervention. Elevated ADP and AA levels indicate excessive platelet activation, which can lead to the release of large amounts of proinflammatory and vasoconstrictive mediators, compromising the integrity of the placental endothelium (15), increasing the thrombotic burden in the microcirculation, and increasing the risk of embryo rejection (16). These findings are consistent with the study by Zeng JZ et al. (17, 18) and support the inclusion of platelet aggregation function in routine first-trimester screening for RSA. For high-risk patients with substantially elevated ADP and AA levels, early administration of antiplatelet interventions such as low-dose aspirin can inhibit excessive platelet activation, improve placental perfusion, and reduce the risk of miscarriage (19).

PT and INR showed no substantial differences between the two groups, suggesting that routine baseline coagulation screening has limited discriminatory power for assessing the risk of RSA recurrence and may be ineffective when used alone for risk prediction in early pregnancy. Thus, conducting a comprehensive assessment by combining these markers with thrombosis-specific anticoagulation, fibrinolysis, and platelet function indicators is recommended.

### 4.2 The independent effect of thyroid dysfunction on pregnancy outcomes in women with RSA

Thyroid disorders are common in women during pregnancy and childbirth. The most common etiology of hypothyroidism during this period is autoimmune thyroiditis, while hyperthyroidism is primarily caused by Graves’ disease and transient gestational thyrotoxicosis. Hypothyroidism and hyperthyroidism during pregnancy and childbirth increase the risk of gestational hypertension, miscarriage, low birth weight, and stillbirth, and can affect the intellectual and neurological development of the offspring. Furthermore, subclinical hypothyroidism during pregnancy and the perinatal period can also lead to adverse pregnancy outcomes. In normal pregnancy, hCG physiologically suppresses pituitary TSH secretion, maintaining moderately reduced TSH levels and stable FT4 levels to meet the developmental needs of the embryo. This study showed that FT4 and TSH levels were substantially higher in the pregnancy failure group than in the pregnancy success group. When combined with its incidence, the prevalence of thyroid disease was substantially higher in the pregnancy failure group than in the pregnancy success group. Although the mean values in both groups were normal for early pregnancy, the differences in incidence and the statistically significant differences in the indicators between the two groups suggest that central thyroid dysfunction—resulting from the disruption in the negative feedback regulation of the hypothalamic–pituitary–thyroid axis—in the pregnancy failure group is associated with pregnancy loss in patients with RSA.

Substantial differences were not observed in FT3 levels between the groups; hence, FT3 has weaker discriminatory power for predicting adverse outcomes of RSA in early pregnancy compared to FT4 and TSH. Therefore, FT4 and TSH may be considered core indicators for thyroid monitoring during the clinical assessment of the RSA risk. Since this study did not include a stratified analysis of thyroid autoantibodies, it could not distinguish whether thyroid dysfunction was autoimmune or purely functional. The associated confounding effects require further validation in subsequent stratified cohorts.

Multivariate logistic regression analysis showed that after adjusting for confounding factors, elevated FT4 and TSH levels were independent risk factors for recurrent pregnancy loss. Thus, thyroid hormones may play a role in processes such as the maintenance of luteal function, the proliferation and differentiation of placental trophoblasts, and the regulation of maternal–fetal immune tolerance (20, 21). However, it should not be overlooked that TSH levels in pregnant women undergo dynamic changes even during early pregnancy (22). Moreover, there is no currently clear, unified standard for these changes.

### 4.3 Interactions between the thrombosis–thyroid axis and variations in pregnancy outcomes

Spearman’s correlation analysis confirmed group-specific differences in the association between thrombotic markers and thyroid function. In the group with successful pregnancies, thrombotic markers such as AT and ADP showed a weak positive correlation with TSH, whereas they exhibited a slight negative correlation in the group with pregnancy failure. Since the difference in the correlation coefficient between AT and TSH across the two groups was statistically significant, the synergistic action of the thrombosis–thyroid regulatory axis may be one of the key factors in maintaining a sustained pregnancy.

In non-pregnant physiological states, low FT4 levels lead to a hypocoagulable and hyperfibrinolytic state, increasing the risk of bleeding. As thyroid hormone levels rise, coagulation increases, fibrinolysis decreases, and the risk of thrombus formation—particularly venous thromboembolism—increases (23). Hypothyroidism may also affect the vascular system through mechanisms such as endothelial dysfunction, hyperhomocysteinemia, systemic inflammation, and platelet abnormalities (24). To date, no studies have clearly established the interrelationship between pregnancy, thrombosis, and thyroid disease. This study conducted only a correlational analysis and cannot determine the causal relationship between thrombotic markers and thyroid dysfunction. Therefore, the interactive pathogenic mechanisms between the two require further support from basic laboratory studies and prospective cohort studies.

### 4.4 Clinical implications of the predictive performance of various indicators

The ROC curve results showed that the AUC values for each included independent risk factor ranged from low to moderate (with PS having the highest AUC of 0.6628 and DD having the lowest AUC of 0.591), indicating that no single marker can accurately distinguish the risk of recurrent miscarriage in patients with RSA. RSA is a disease mediated by multiple factors, including genetic, anatomical, immunological, metabolic, and coagulation factors. Hence, it is indeed difficult for a single laboratory indicator to fully cover all pathogenic pathways.

From a clinical practice perspective, relying solely on FT4, TSH, or individual coagulation or platelet markers to screen high-risk RSA populations is prone to missed diagnosis or misdiagnosis. In contrast, the combined testing of PS, DD, ADP, AA, FT4, and TSH provides multidimensional coverage of coagulation, platelet activation, and thyroid regulation, thereby improving the sensitivity and specificity of risk identification in early pregnancy and providing an objective basis for stratified interventions.

### 4.5 Limitations of the study

This study has several substantial limitations. First, it was a single-center, retrospective study, with cases drawn from a single source, resulting in limited population representativeness and selection bias. Therefore, causal relationships cannot be inferred. The results can only be used to verify the statistical association between the indicators and pregnancy outcomes. Second, confounding variables such as thyroid autoantibodies, blood glucose levels, and progesterone supplementation were not collected. Multivariate models cannot fully eliminate the interference of these factors, and residual confounding is difficult to avoid. Third, the study included only a single test result from early pregnancy and did not conduct repeated dynamic testing throughout gestation; thus, reflecting the impact of changes in these indicators on pregnancy outcomes as gestational age progresses is impossible. Fourth, with a sample size of only 400 cases, stratified analysis was not performed by miscarriage subtype (biochemical pregnancy, early miscarriage, and mid-term miscarriage), and differences in indicator characteristics across different miscarriage phenotypes remain unclear. Fifth, a precise combined predictive model was not developed, and the universality of the cutoff values for each indicator requires validation through multicenter, large-sample prospective studies.

## 5 Conclusion

In summary, elevated PS levels, increased platelet aggregation (reflected by increased ADP and AA), elevated FT4 and TSH levels, and reduced DD may be independent risk factors for recurrent pregnancy loss in patients with RSA. There is a certain degree of correlation between thrombotic and thyroid function, which may be one of the causes of pregnancy loss. Accordingly, we recommend combined testing of seven core indicators—coagulation and fibrinolysis, platelet function, and thyroid function—during early pregnancy to enable early risk identification, with the hope of improving pregnancy outcomes for patients with recurrent miscarriage. However, given the retrospective design, limited sample size, limited cumulative miscarriage events, and potential residual confounding factors, these trend-based findings should be considered only as directional guidance. Larger-scale prospective studies are necessary to validate these findings.

## Data Availability

The raw data supporting the conclusions of this article will be made available by the authors, without undue reservation.
